# Identification of driver genes based on gene mutational effects and network centrality

**DOI:** 10.1186/s12859-021-04377-0

**Published:** 2021-09-24

**Authors:** Yun-Yun Tang, Pi-Jing Wei, Jian-ping Zhao, Junfeng Xia, Rui-Fen Cao, Chun-Hou Zheng

**Affiliations:** 1 Key Lab of Intelligent Computing and Signal Processing of Ministry of Education, College of Computer Science and Technology, Anhui University, Hefei, China; 2 Institute of Physical Science and Information Technology, Anhui University, Hefei, China; 3 Engineering Research Center of Big Data Application in Private Health Medicine, Fujian Province University, Putian, Fujian China; 4 College of Mathematics and System Sciences, Xinjiang University, Urumqi, China

**Keywords:** Cancer, Driver genes, Mutation data, Local centrality, Transcriptional network

## Abstract

**Background:**

As one of the deadliest diseases in the world, cancer is driven by a few somatic mutations that disrupt the normal growth of cells, and leads to abnormal proliferation and tumor development. The vast majority of somatic mutations did not affect the occurrence and development of cancer; thus, identifying the mutations responsible for tumor occurrence and development is one of the main targets of current cancer treatments.

**Results:**

To effectively identify driver genes, we adopted a semi-local centrality measure and gene mutation effect function to assess the effect of gene mutations on changes in gene expression patterns. Firstly, we calculated the mutation score for each gene. Secondly, we identified differentially expressed genes (DEGs) in the cohort by comparing the expression profiles of tumor samples and normal samples, and then constructed a local network for each mutation gene using DEGs and mutant genes according to the protein–protein interaction network. Finally, we calculated the score of each mutant gene according to the objective function. The top-ranking mutant genes were selected as driver genes. We name the proposed method as mutations effect and network centrality.

**Conclusions:**

Four types of cancer data in The Cancer Genome Atlas were tested. The experimental data proved that our method was superior to the existing network-centric method, as it was able to quickly and easily identify driver genes and rare driver factors.

## Background

Cancer is one of the most complex diseases that threaten human health [1]. The latest developments in next-generation sequencing (NGS) technology have provided us with an unprecedented opportunity to better characterize the molecular characteristics of human cancer [2, 3]. The Cancer Genome Atlas (TCGA) [4] and the International Cancer Genome Consortium (ICGC) [5] have produced and analyzed a large amount of genomic data of various cancers [6]. Cancer development involves many complex and dynamic cellular processes. These processes can be accurately described according to the pathological stages, and the extraction of reliable biomarkers is required to characterize the dynamics of these stages, including (1) stage-specific recurrence somatic copy number alterations (SCNAs), (2) the related aberrant genes, and (3) the enriched dysfunctional pathways [7–12]. The key challenge for cancer genomics is analyzing and integrating this information in the most efficient and meaningful way, which can promote cancer biology and then translate this knowledge into clinical practice [13, 14]; for example, the design of anticancer drugs and identification of drug-resistant genes [15]. Cancer is an evolutionary process in which normal cells accumulate various genomic and epigenetic changes, including single-nucleotide variations (SNVs) and chromosomal aberrations. Some of these alterations give mutant cells an advantage in growth and positive selection as well as cause intense proliferation, giving raise to tumors [16]. Although somatic mutations occur in normal cells, they are neutral or apoptosis-inducing, not leading to conversion to cancer cells [17]. One of the key questions in cancer genomics is how to distinguish ‘driver’ mutations that cause tumors from ‘passenger’ mutations that are functionally neutral [18].

The simplest way to identify driver genes is to classify mutations according to recurrence; in other words, the most frequently occurring mutations are more likely to be drivers [19, 20], or the background mutation rates are used to measure significantly mutated genes. Many computational methods based on mutation frequency recognition for driver mutations and driver genes have been widely used, such as MutSig [21] and MuSic [22]. MuSig estimates the background mutation rate of each gene and identifies mutations that deviate significantly from that rate. MuSic uses mutation rates that are significantly higher than expected, pathway mutation rates, and correlations with clinical features to detect driver genes. Tamborero et al. used a silent mutation in the coding region to construct a background model and proposed the OncodriveCLUST method, which is mainly used to identify genes with a significant mutation clustering tendency in protein sequences [23]. However, a portion of the driver genes are mutated at high frequencies (> 20%), and most cancer mutations occur at intermediate frequencies (2–20%) or lower frequencies than expected [24]. Although frequency-based methods can identify driver genes among genes that are frequently mutated in patients, they are ineffective in identifying drivers in infrequently or rarely mutated genes [25]. To obtain sufficient statistical power to detect cancer driver genes with low mutation frequency, a large number of cancer patients must be sequenced [26]. This situation has provoked a number of methods that assist in identifying driver genes. Generally, these methods can be categorized into machine learning-based methods and network-based methods.

Machine learning-based approaches use existing knowledge to identify driver genes or driver mutations. For example, CHASM uses random forests to classify driver mutations and uses known carcinogenic somatic cells for missense mutation training [27]. Moreover, the CHASM score has also been successfully applied to the CRAVAT algorithm [28]. In addition to CHASM, the CRAVAT algorithm integrates the results of the SNVBox [29] and VEST [30] tools and realizes the annotation of the effect of non-synonymous mutation functions [28]. The CanDrA algorithm integrates the results of more than 10 algorithms (such as CHASM, SIFT, and MutationAssessor); obtains 96 features in structure, evolution, and genes; and builds an algorithm based on machine learning prediction-driven missense mutations [31]. The FATHMM algorithm integrates homologous sequences and conserved protein domain information and uses a hidden Markov model-based algorithm to distinguish cancer-related amino acid mutations among passenger mutations [32, 33]. The DriverML algorithm proposed by Han et al. used statistical methods to quantify the scores of different mutation types on protein function and then combined them with machine learning algorithms to identify cancer driver genes [34]. However, the method of training prediction models using machine learning has some shortcomings. For example, in predicting driver mutations, it is difficult to obtain high-quality positive and negative sample datasets, which is a significant challenge for machine learning-based algorithms.

The development of network analysis science, such as in the fields of complex systems, social networks, communication networks, and transportation networks, has inspired many bioinformatics researchers to use network analysis methods to study the functional mechanism of molecular systems. Pathway- and network-based methods can easily simplify biological entities and their interactions into nodes and edges, allowing the systematic study of the nature of complex diseases [35] and the diagnosis, prevention, and treatment of cancer. Moreover, network- and pathway-based strategies have become one of the most promising approaches for identifying driver mutations, and some researchers have found that genes work together to form biological networks, which can be used to identify driver genes. MEMO [36] relies on the predictive pathway or the mutual exclusion of driving mutations in the sub-net to try to find a small sub-net of genes belonging to the same pathway. PARADIGM-Shift [37] uses pathway-level information and other features to infer the dysfunction of mutations. Researchers have also attempted to use protein–protein interaction network (PPI) data to integrate different omics data. For example, HotNet2 [38] combined with PPI used hotspot diffusion to find the small sub-networks of frequent mutations. However, the authors tried to identify a cancer-driving module composed of many genes, rather than genes that are crucial for cancer development. A recently published method, DriverNet [39], identifies a simple set of mutated genes associated with genes that experience mRNA expression disorders in a PPI network. OncolMPACT [40] prioritizes mutated genes based on linkages to dysregulated genes in cancer using matched expression data. The VarWalker algorithm, through sample-specific gene screening, constructs a sample-specific network, and integrates and recognizes driver genes [41]. The DawnRank algorithm analyzes the effect of a mutant gene on its downstream genes in a molecular interaction network, and used the PageRank algorithm sequences the genes of a single sample, finally resulting in the identification of driver genes [3]. The DEOD algorithm integrates genomic mutation data, expression data, and PPI network data; constructs a directed weighted graph based on the method of partial covariance selection; and identifies driver genes that have a significant effect on the target gene [42]. MUFFINN [43] considers mutations in neighboring genes in a network in two different ways, either consider mutations in the most frequently mutated neighbor (DNmax) or to consider mutations in all direct neighbors with normalization by their degree connectivity (DNsum) showing good predictive performance in large candidate sets.

In recent years, researchers have also attempted to identify driver genes from the perspective of individual networks. For example, the SSN algorithm is based on individual network identification of driver genes, which uses the Pearson Correlation Coefficient (PCC) of sample expression data to construct individual networks and then, through statistical analysis, determine cancer driver genes or modules [44]. The HIT’n DRIVE algorithm integrates each patient’s individual genomic mutation data and expression data to construct a network and identify the driver genes and modules that affect transcriptional changes based on the expected value of the shortest random walk length in the network [45]. From the perspective of individuals, Guo et al. successively proposed the SCS [46] and PNC [47] algorithms. The SCS algorithm integrates mutation data, expression data, and molecular network data of each patient sample, and uses the network control method to evaluate the individual genes. Driver genes are then identified based on the effect of gene mutations on gene expression [46]. The PNC algorithm uses paired samples to construct individual networks, and then uses structure-based network control principles to identify individual driver genes [47]. The PRODIGY algorithm proposed by Dinstag et al. integrates individual mutation and expression data with pathways and PPI network data, uses reward collection Steiner tree models to quantify the regulatory effects of mutant genes on pathways and recognize driver genes [48]. However, owing to incomplete data in gene interaction networks, the false positive rate of these existing methods is still very high; therefore, further improvement is needed, which brings challenges to network-based prediction methods.

To overcome false positives and improve prediction accuracy, in this study, we introduced semi-local centrality and considered mutational information between genes to identify mutant genes in tumors. Unlike DriverNet, we considered the structure of the genes in the network. The introduction of network centrality can lead to the identification of genes at key locations in the network. These genes may be driven by genes or regulatory genes. MUFFINN considers the direct neighbor information of mutated genes in the network, but ignores the information of the secondary neighbor. Based on this, our method considered not only the nearest and the next-nearest neighbors of node but also the interaction between mutant gene nodes. We processed the cancer coding region mutation data from TCGA into a gene–patient mutation matrix as well as calculated the gene mutation score and the Euclidean distance between two genes according to the matrix. Increasing evidence shows that miRNAs are widely involved in the occurrence of cancer [49, 50]; therefore, we also performed gene expression analysis to obtain differentially expressed genes. Moreover, functional studies have suggested that driver mutations alter the expression of its downstream genes in the molecular interaction network [51]; therefore, we integrated differentially expressed genes and mutated genes into the PPI network and calculated the effect of the mutated genes based on the obtained local network. Experiment on TCGA datasets verified that our proposed mutations effect and network centrality (MENC) method was superior to the existing methods based on frequency and network centrality.

## Results

Most existing network methods for identifying driver genes are based on global networks. These global networks increase computational complexity. In addition, the accuracy of these methods needs to be improved. Our method employed a novel scheme: we first calculated the effect of the mutation, and then identified a local network for each mutated gene. We used the objective function to calculate the effect of mutated genes in the local network and sort the mutated genes according to the score to determine the driver genes. The top-ranking genes were more likely to become driver genes, which are more interesting to researchers and can even advance to further biological experiments for verification. Therefore, in the comparison analysis, we only used the top 50 candidate genes. To show the advantages of our model, we analyzed four large-scale publicly available datasets, including glioblastoma (GBM), bladder cancer (BLCA), prostate cancer (PRAD), and ovarian cancer (OVARIAN). The experimental results showed that our method was better than not only the network-centric method but also other types of methods. More importantly, our method was also able to recognize rare driver genes.

### Datasets and resources

In this study, we mainly used two types of data: coding region mutation data and gene expression data. In particular, the coding region mutation data included copy number variations (CNVs) and SNVs. These data were obtained from 328 GBM samples, 379 BLCA samples, 252 PRAD samples, and 316 OVARIAN samples, and downloaded from the TCGA data portal (https://tcga-data.nci.nih.gov/tcga/). We used only samples that included both of them. The PPI network we used was downloaded from the Human Protein Reference Database (HPRD) [52, 53], which consists of 9617 genes and 74,078 edges. Table 1 shows the sample counts in the four cancers mapped on the PPI network mutated gene numbers and outlying gene numbers.

**Table 1:** Description of datasets

Tumor type	Number of tumor expression samples	Number of mutation samples	Map to DEGs on the network	Map to mutation genes on the network
GBM	328	328	4196	5650
BLCA	379	379	8787	8029
PRAD	252	252	5953	4184
OVARIAN	316	316	5309	5705

In the absence of basic facts, quantitative measurements using standard sensitivity/specific benchmarking techniques are impractical. To help assess the quality of our results, we obtained a list of 616 known drivers from the Cancer Gene Census (CGC) database (09/26/2016) [54].

### Comparison with network-centric approaches

To evaluate the method’s ability to identify known driver genes, we compared our method with network centricity-based methods. As mentioned above, we used the CGC as an approximate benchmark for known driver genes. For comparison, we used the following three metrics (precision and recall rates and F1score) in this study:

1 $$\begin{aligned} & Precision = \frac{{(\# {\text{Mutated genes in CGC}}) \cap (\# {\text{Genes found in MENC}})}}{{(\# {\text{Genes found in MENC}})}} \\ & {\text{Recall}} = \frac{{(\# {\text{Mutated genes in CGC}}) \cap (\# {\text{Genes found in MENC}})}}{{(\# {\text{Genes found in CGC}})}} \\ & {\text{F1score}} = 2 \times \frac{Precision \times Recall}{{Precision + Recall}} \\ \end{aligned}$$

**Fig. 1:**
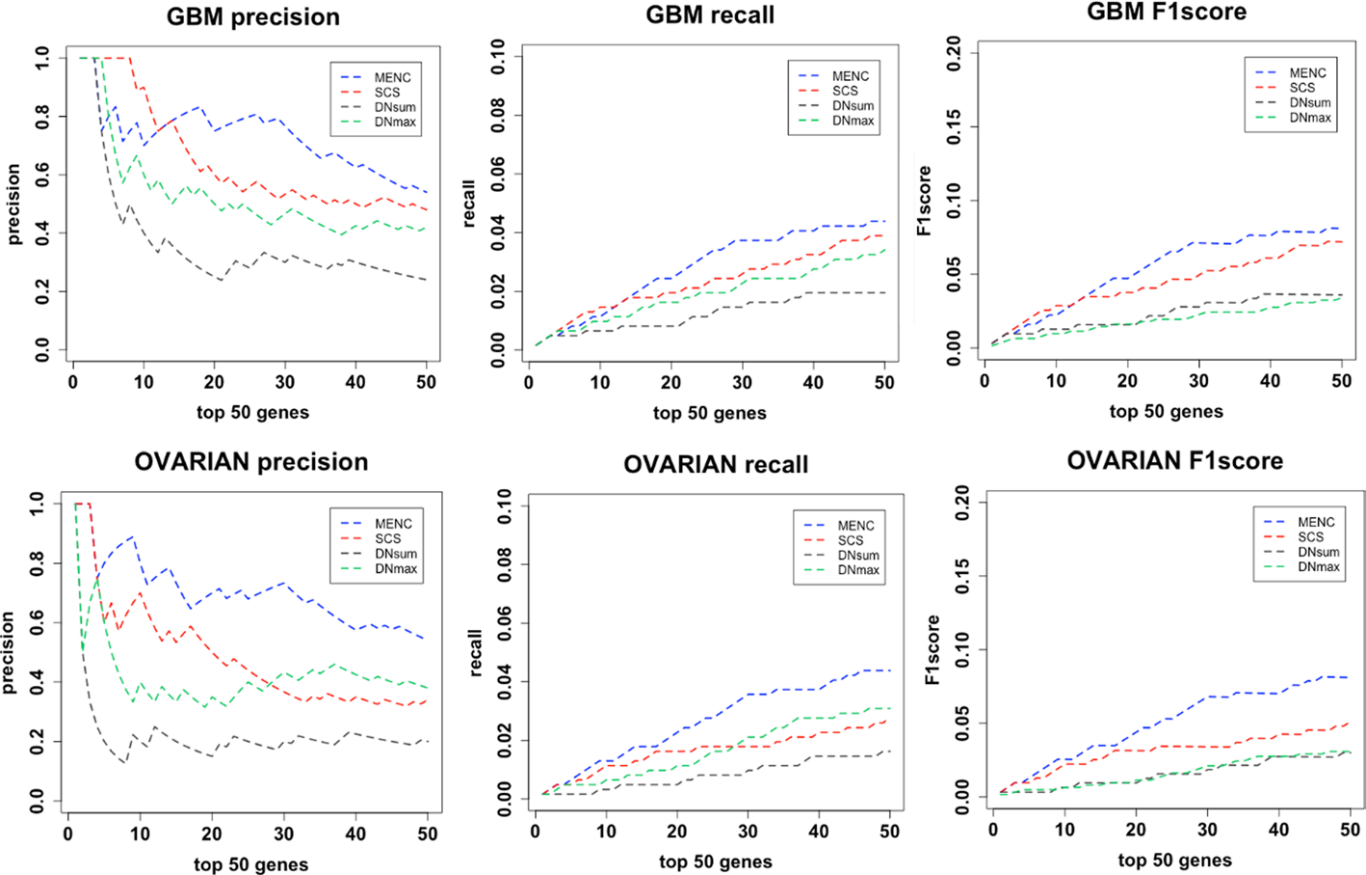
Comparison of precision, recall, and F1score for the top-ranking genes. The *X* axis represents the number of top-ranking genes, and the *Y* axis represents the score of the precision, recall, and F1score

We compared our method with two main network-centrality-based methods, SCS [46] and MUFFINN [43]. MUFFINN considers mutational information among direct neighbors, either in the most frequently mutated neighbor (DNmax) or in all direct neighbors with normalization by their degree of connectivity (DNsum). The results are shown in Fig. 1. Here, we only show the results for two types of cancer (GBM and OVARIAN). As shown in the figure, our method performed better than SCS and MUFFINN. For GBM cancer, our method was not as effective as SCS in identifying the first 15 candidate driving genes, but our method showed a great improvement in the latter. MENC was significantly superior to the other methods for the other three cancers. The number of CGCs covered among the top 50 genes identified was 27 genes with our method, 24 with SCS, 12 with DNsum, and 21 with DNmax. Our method achieved the best results for the BLCA and PRAD cancer data.

For OVARIAN cancer, the top 50 genes analyzed by our method included 27 in the CGC database, while SCS had 17, DNsum had 10, and DNmax had 17. It can also be seen that the SCS method exhibits a large downward trend. The accuracy of the top 10 genes was 0.8, and the accuracy was reduced to below 0.4 in the top 30. Our method is relatively stable, and there is no significant decline. The results indicated that our method yielded reliable results for identifying driver genes.

### Comparison with other approaches

**Fig. 2:**
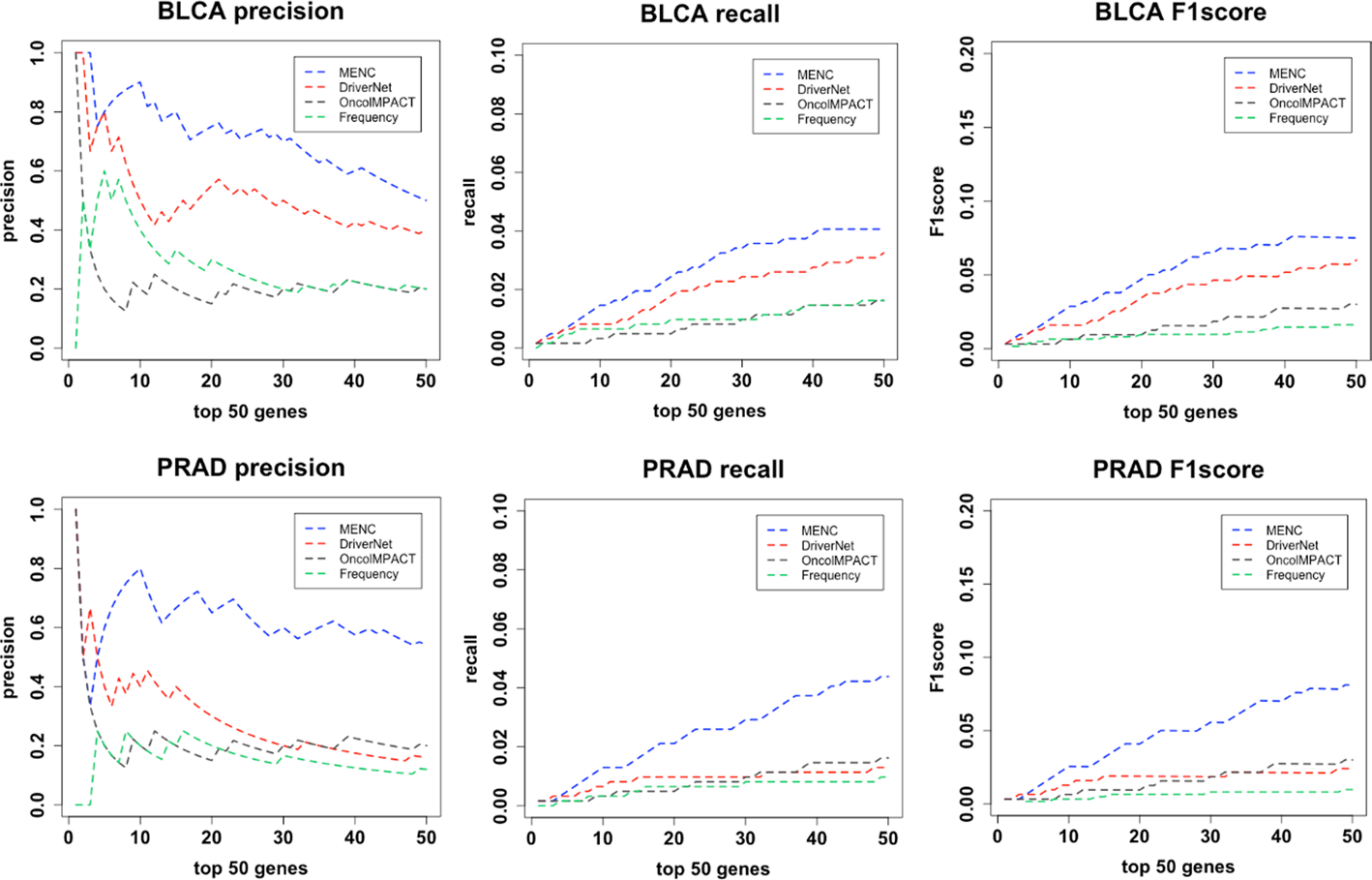
The comparison of precision, recall and F1score for top ranking genes of MENC and other methods. The *X* axis represents the number of top ranking genes and the *Y* axis represents the score of the precision, recall and F1score respectively. The last row is the result of BLCA and the next row is the result of PRAD

Because our method not only considers the characteristics of the network but also calculates the mutation scores and interaction of the genes, we also compared MENC with DriverNet [39], a frequency-based method, and OncolMPACT [40]. As shown in Fig. 2, in general, relative to CGC, our approach was superior to DriverNet, Frequency, and OncolMPACT in analyzing all cancer datasets. Although only the results of BLCA and PRAD cancers are shown here, the same good results were obtained for other cancer data, which are not shown here.

### Novel and reliable driver genes found using our method

In addition to identifying frequently mutated driver genes, MENC can identify important rare driver genes. According to DawnRank’s [3] definition of novel and important driver genes, genes meeting the following requirements are rare genes: (1) the ranking of the driver gene is based on patient population; (2) frequency of the mutation is less than 2% of the patient population in the mutation data; (3) the gene has not been identified as a driver gene by CGC.

In OVARIAN, 316 samples were analyzed. Using our method, nine rare driving factors were identified as the top 20 genes according to the above definition, seven of which were in included in CGC (see Table 2). Although some rare driver genes such as EGFR, EP300, and CREBBP have been found in DNMax and DNSum, they rank higher in our method. In addition, SRC (1.58% of cases) is usually associated with disease and may lead to the development of human malignancies [55]. FYN (0.95% of cases) and PRKCA (1.58% of cases) have not been listed as driving genes by CGC, but studies have found that they are associated with many cancers and overexpressed in cancer patients [56, 57].

**Table 2:** Rare driver genes in OVARIAN

Rank	Gene	Mut	Mutation frequency (%)	CGC gene
2	*SRC*	5	1.582278	YES
8	*EP300*	6	1.898734	YES
9	*SMAD3*	2	0.632911	YES
11	*FYN*	3	0.949367	NO
12	*PIK3R1*	6	1.898734	YES
13	*AR*	1	0.316456	YES
17	*PRKCA*	5	1.582278	NO
19	*PTPN11*	6	1.898734	YES
20	*SMAD4*	4	1.265823	YES

In BLCA, we identified 18 rare genes among 22 candidate driver genes (see Table 3), 12 of which were in CGC. For example, MENC recognized AKT1 (0.53% of cases) as a serine/threonine protein kinase, and its downstream proteins have been reported to be frequently activated in human cancers [58]. Most of the highest-ranked genes in BLCA are low-frequency mutant genes.

**Table 3:** Rare driver genes in BLCA

Rank	Gene	Mut	Mutation frequency (%)	CGC genes
2	*SRC*	4	1.055409	YES
3	*ESR1*	1	0.263852	YES
4	*GRB2*	1	0.263852	NO
8	*MAPK1*	2	0.527704	YES
9	*AR*	2	0.527704	YES
10	*PIK3R1*	3	0.791557	YES
11	*SHC1*	4	1.055409	NO
12	*SMAD3*	5	1.319261	YES
13	*FYN*	3	0.791557	NO
14	*ABL1*	7	1.846966	YES
15	*SMAD2*	3	0.791557	YES
16	*PRKCA*	3	0.791557	NO
17	*CSNK2A1*	5	1.319261	NO
18	*STAT3*	6	1.583113	YES
19	*LCK*	1	0.263852	YES
20	*BRCA1*	4	1.055409	YES
21	*AKT1*	2	0.527704	YES
22	*PRKCD*	1	0.263852	NO

**Fig. 3:**
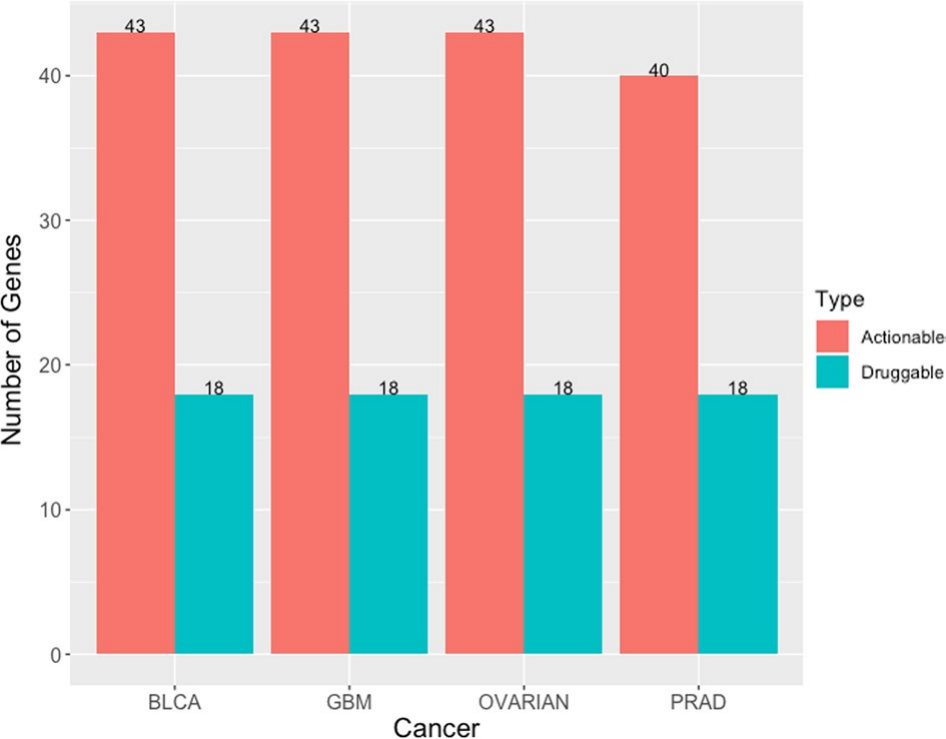
Actionable and druggable genes among candidate driver genes in four types of cancer

Considering that the identification of cancer driver genes is required for cancer treatment, we used the drug–genes interaction database (DGIdb) [59] and TARGET database [60] to determine whether our candidate driver genes are clinically relevant genes. The results are shown in Fig. 3. In all four cancer datasets, 80% or more candidate driver genes were identified as actionable targets. Approximately 40% of the genes were druggable. There is a partial intersection between the candidate genes and druggable genes. The union of the actionable and druggable genes in the four cancers BLCA, GBM, OVARIAN, PRAD was 42, 42, 39, and 42, respectively. These results indicate that the candidate driver genes are clinically relevant.

### Enrichment analysis

To test the biological function of the MENC-predicted candidate drivers, we used the DAVID tool (v6.8) for KEGG pathway and GO function enrichment analyses.

For OVARIAN, the important candidates were mainly enriched in pathways in cancer, viral carcinogenesis, proteoglycans in cancer, prostate cancer, and pancreatic cancer. They were also involved in biological process such as positive regulation of transcription from RNA polymerase II promoter and signal transduction. Regarding cellular components, the identified candidates were enriched in the nucleus, nucleoplasm, cytosol, cytoplasm, and plasma membrane. Furthermore, with regards to important molecular functions, the candidate drivers were enriched in identical protein binding, DNA binding, and transcription factor binding.

In BLCA, KEGG analysis showed that the candidate genes were enriched in pathways in cancer, chemokine signaling pathway, and PI3K-Akt signaling pathway. GO analysis revealed that the candidate genes were enriched in signal transduction, positive regulation of transcription, and DNA template. As for cellular components, the candidates were enriched in the cytoplasm and nucleus. In terms of molecular functions, the candidates were enriched in protein binding, enzyme binding, and transcription factor activity.

In GBM, the candidates were enriched in pathways in cancer, viral carcinogenesis, and hepatitis B. In terms of biological processes, the candidate drivers were enriched in signal transduction, viral processes, and protein phosphorylation. With respect to cellular components, the candidates were enriched in the nucleus, plasma members, cytoplasm, and nucleoplasm. As for molecular functions, the candidates were enriched in enzyme binding, transcription factor activity, and sequence-specific DNA binding.

In PRAD, the enriched KEGG pathways were proteoglycans in cancer, thyroid hormone signaling pathway, and microRNAs in cancer. The enriched GO functions were negative regulation of the apoptotic process and protein phosphorylation. As for cellular components, the candidates were enriched in the cytosol, nucleus, and plasma membrane. In terms of molecular functions, the candidate drivers were enriched in protein binding, ATP binding, transcription factor binding, and kinase activity.

## Discussion and conclusions

In this study, we proposed the MENC method for identification of driver genes. Our approach not only considered mutation frequency in patients but also integrated mutation and gene expression data into a gene–gene interaction network. We considered the nearest and next-nearest nodes from the source node when calculating the network centrality. When tested on the GBM and OVARIAN datasets, our method performed significantly better than the network-based SCS and MUFFIN methods. In addition, our method was superior to other methods such as DriverNet in analyzing the PRAD and BLCA datasets. Our method even identified rare driver genes.

Nevertheless, our approach had some limitations. For example, in clinical practice, precision medicine and personalized medicine are important for the diagnosis and treatment of patients. However, using the proposed method, we could not diagnose driver genes in the individual. In the future, we will propose a new approach to identify patient-specific and rare driver genes based on individual mutations and gene expression profiles in tumors.

## Methods

### Overview of the MENC approach

**Fig. 4:**
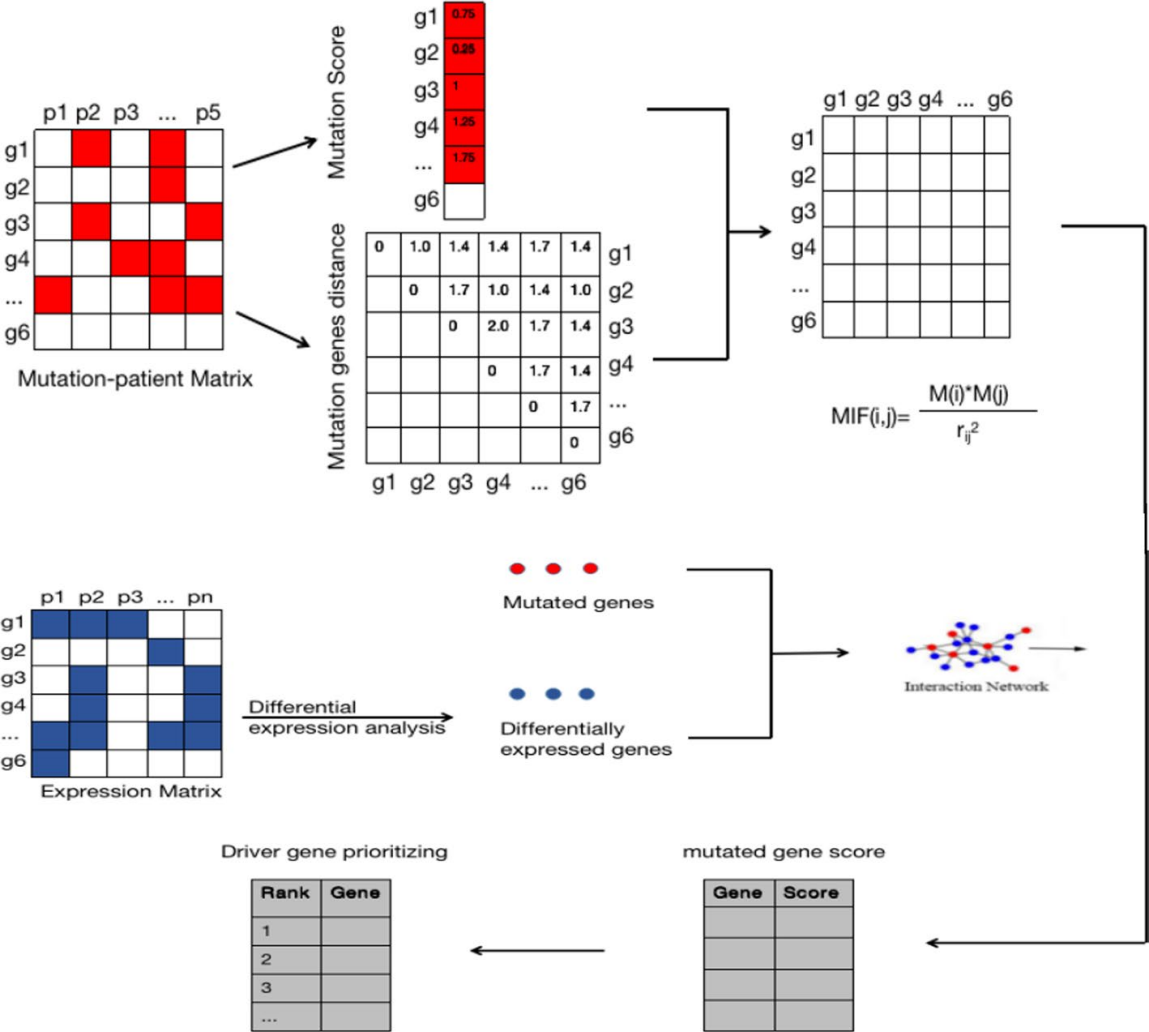
Flowchart of comparative transcriptome analysis of the mutations effect and network centrality (MENC) method used in this study. The red nodes represent the mutated gene from the mutation-patient matrix, and the blue nodes represent the differentially expressed genes from the gene expression matrix

We proposed a new method that combined mutation and expression data into a PPI network, and adopted a combination of semi-local centrality and mutation effect function to identify the driver genes of cancer. The method consisted of three main steps. First, we integrated SNV and CNV data to obtain a mutation matrix, and calculated the gene mutation score (Eq. 2) and the Euclidean distance (Eq. 3) between two genes according to the matrix. Next, the mutation effect function between genes was calculated according to Eq. 4. In the second step, we compared the expression profiles of tumor samples with those of normal samples to identify DEGs. We subsequently constructed a semi-local network for each mutation gene using DEGs and mutation genes according to the PPI network. The third step was to calculate the local centrality and mutation effect of the mutated genes according to the target function (Eq. 5). The top-ranking genes were regarded as candidate driver genes. Our method considered the nearest and next-nearest nodes when calculating the local centrality. Compared with global centrality measures (e.g., betweenness centrality and closeness centrality), our local centrality measure had a much lower computational complexity. We also added the mutational effect function, as to not ignore some genes that have a low degree but may have a much higher influence than high-degree genes [61]. A flowchart of the method is shown in Fig. 4.

### Calculation of gene mutation score and distance between genes

The downloaded TCGA coding region mutation data were summarized in a binary gene-patient matrix *M*, in which the rows represent the genes, and the columns represent the cancer samples (patients). For gene *i*, if the patient has SNVs or CNVs, *M*(*i*, *j*) = 1; otherwise, *M*(*i*, *j*) = 0. We used the MaxMIF [62] method to calculate the mutation score (Eq. 2). Based on the obtained gene-patient matrix, we calculated the mutation score of the gene. The mutation score *M*(*i*) for each gene *i* accounts for the contribution of its mutation to cancer, defined as follows:

2 $${\text{M}}({\text{i}}) = \left\{ {\begin{array}{*{20}l} {\sum\nolimits_{{k \in K_{i} }} {\frac{1}{{N_{k} }}} ,} \hfill & {\quad K_{i} \ne \Phi } \hfill \\ {\frac{1}{{N_{\max } }},} \hfill & {\quad K_{i} = \Phi } \hfill \\ \end{array} } \right.$$

where *K*_*i*_ is the set of patients with mutations in gene *i*. *N*_*k*_ is the total number of mutated genes in sample *k*. *N*_*max*_ is the maximum number of mutated genes in all samples. If gene *i* has no mutation in all samples, that is, *K*_*i*_ is empty, then *M*(*i*) is assigned a background mutation score (BMS) that is no greater than any mutant gene.

We then calculated the Euclidean distance between two genes according to the distance formula (Eq. 3), where vector *X*, *Y* is the row vector of each gene in the gene-patient matrix, and *x*_*i*_, *y*_*i*_ is an element in the row vector. In this study, we also tried other distance formulas, such as Jaccard and Manhattan, and brought the distance obtained by each distance formula into the final objective function. We found that the obtained driver genes were the same; therefore, we chose the Euclidean distance in the experiment.

3 $${\text{dist}}(X,Y) = \sqrt {\sum\limits_{i = 1}^{n} {(x_{i} - y_{i} )^{2} } }$$

### Mutations effect function between genes

Reference MaxMIF measures the effect of interaction between two mutant genes on biological functions. In this experiment, we also used mutation impact function (MIF) values to calculate the effect of mutation between two genes. The value is driven by the gravity principle [63].

4 $${\text{MIF}}(i,j) = \frac{M(i)M(j)}{{r_{ij}^{2} }}$$

Here, *M*(*i*) and *M*(*j*) are the mutation scores of gene *i* and *j*, respectively. *r*_*ij*_ is the reciprocal of the Euclidean distance between gene *i* and gene *j*. Euclidean distance measures the similarity of two vectors (the similarity of two genes on the patient set). Two genes with high mutation scores and high similarity had high MIF values.

### Identification of DEGs and construction of local network

In this study, expression data were processed the same way as SCS data. To indicate the DEGs of each patient, we first calculated the log2 fold-change in gene expression between the paired tumor and normal samples. Genes with an absolute value greater than 1 were considered as DEGs. We then collected the DEGs from each patient to obtain the DEGs of the cohort. All patient mutation genes were selected from the mutation matrix. In addition, we downloaded the PPI network as an interaction graph between the mutated genes and DEGs. If there are edges of mutant genes and DEGs in the network, the two genes are connected to the semi-local network. We built a semi-local network where mutated genes were considered the source node and DEGs were the target nodes. Moreover, we only considered the role of the mutant in two steps, which reduced the computational complexity. After preprocessing the data, the next step was performed.

### Calculation of driver gene scores

Unlike some existing network-based methods, we constructed a new semi-local intersection network for each mutated gene by merging mutant genes, DEGs, and HPRD networks. Referring to the metric of the network local centrality measure *C*_*L*_(*v*) in [61], *C*_*L*_(*v*) calculates the number of neighbors of node *v* and the neighbors of the neighbors. We have made corresponding improvements to this formula: when counting the number of neighbors of a node gene, we performed different calculations for the neighbors of the node that were mutations and DEGs. If the neighbor of the node was a mutated gene, we used the MIF between the genes multiplied by the degree of the node, and if the neighbor was the DEGs, only the degree of the node was calculated. See formula (5):

5 $$\begin{aligned} score(v) & = N(v) + \sum\limits_{\begin{subarray}{l} u \in N(u) \\ u \in Mutation \end{subarray} } {c(u)*MIF(v,u)} + \sum\limits_{\begin{subarray}{l} u \in N(u) \\ u \in DEGs \end{subarray} } {b(u)} \\ c(u) & = \sum\limits_{\begin{subarray}{l} w \in N(u) \\ w \in Mutation \end{subarray} } {N(u)} *MIF(u,w) + \sum\limits_{\begin{subarray}{l} w \in N(u) \\ w \in DEGs \end{subarray} } {N(w)} \\ b(u) & = \sum\limits_{\begin{subarray}{l} w \in N(u) \\ w \in Mutation \end{subarray} } {c(w)} + \sum\limits_{\begin{subarray}{l} w \in N(u) \\ w \in DEGs \end{subarray} } {N(w)} \\ \end{aligned}$$

where *N*(*v*)/*N*(*w*) represents the set of neighbors of node *v*/*w*. We calculated the local centrality of the mutated gene. For mutation *i*, if the mutated gene *u*/*w* was ligated, we also considered the mutation effect between them as a weight, calculated by *c*(*u*)/*c*(*w*). Therefore, we can identify drivers that are important in the network and have a strong effect on other genes. If the neighbor *u*/*v* is a DEG, calculated by *b*(*u*)/*b*(*w*), which only considered the centrality of the network. Our main idea was to accord the function as the effect score in a local network. The higher the score, the greater the effect of the mutated gene on the DEGs in the local network. The presence of genes is both a mutation and a differential expression. Therefore, these genes may be more important. Therefore, when a gene is differentially expressed, it acts as a target node. However, when mutated, it acts as a source node. The score for this type of gene increased. Using this model, we obtained a score for each mutant gene. Then, according to the scores, we ranked the mutation genes to identify influential genes. We assumed that the higher the ranking, the more likely it was to be a driver gene.

## Data Availability

All datasets analyzed in the current study were downloaded from the TCGA data portal (https://tcga-data.nci.nih.gov/tcga/). The evaluation data set used was from the CGC gene list of the COSMIC database (https://cancer.sanger.ac.uk/cosmic), version number (09/26/2016).

## Abbreviations

DEGs: Differentially expressed genes
PPI: Protein–protein interaction
MENC: Mutations effect and network centrality
NGS: Next-generation sequencing
TCGA: The cancer genome atlas
ICGC: International cancer genome consortium
SCNAs: Somatic copy vumber alterations
SNVs: Single nucleotide variants
PCC: Pearson correlation coefficient
GBM: Glioblastoma
BLCA: Bladder cancer
PRAD: Prostate cancer
OVARIAN: Ovarian cancer
CNVs: Copy-number variations
HPRD: Human protein reference database
CGC: Cancer gene census
DNmax: Direct neighbor max
DNsum: Direct neighbor sum
DGIdb: Drug–genes interaction database
TARGET: Tumor alterations relevant for genomics-driven therapy
DAVID: Database for annotation, visualization and integrated discovery
BMS: Background mutation score
MIF: Mutation impact function
